# Extramedullary Hematopoiesis in the Appendix Associated With Myelodysplastic Syndrome: A Case Report

**DOI:** 10.7759/cureus.79560

**Published:** 2025-02-24

**Authors:** Naoki Kinjo, Matsukuma Satoshi, Atomu Suzuki, Koji Yamashita, Yoshimi Yamashita

**Affiliations:** 1 Surgery, Japan Community Healthcare Organization (JCHO) Tokuyama Central Hospital, Shunan, JPN; 2 Gastrointestinal Surgery, Japan Community Healthcare Organization (JCHO) Tokuyama Central Hospital, Shunan, JPN; 3 Hematology, Japan Community Healthcare Organization (JCHO) Tokuyama Central Hospital, Shunan, JPN; 4 Pathology, Japan Community Healthcare Organization (JCHO) Tokuyama Central Hospital, Shunan, JPN

**Keywords:** appendix, emh, laproscopic appendectomy, myelodysplastic syndrome (mds), nhs-emh

## Abstract

Extramedullary hematopoiesis (EMH) is characterized by the presence of hematopoietic tissue outside the bone marrow, more commonly in the liver and the spleen. However, there are very few reports of nonhepatosplenic extramedullary hematopoiesis (NHS-EMH). Among the NHS-EMHs, NHS-EMH in the appendix is extremely uncommon. We hereby present a case of appendiceal NHS-EMH and discuss it with a literature review.

The patient was a gentleman in his 70s who had been treated for follicular lymphoma seven years ago. He gradually developed leukopenia and six months ago was diagnosed with myelodysplastic syndrome (MDS). Four days prior to admission, he developed abdominal pain that was localized to the right lower quadrant and was accompanied by fever and loss of appetite. Abdominal CT revealed a thickening of the appendiceal wall and increased fat density around it. A diagnosis of acute appendicitis was made, and he was treated conservatively. However, his condition did not improve, and a laparoscopic appendectomy was performed.

Diffuse proliferation of hematopoietic cells in the submucosal layer of the appendix was observed on histopathological examination. This confirmed the diagnosis of NHS-EMH. The postoperative course was uneventful; however, the patient’s MDS progressed to acute leukemia in the next four months, and he died 14 months after surgery.

Appendiceal NHS-EMH is an extremely rare condition associated with clinical features that are similar to those of acute appendicitis, making preoperative diagnosis challenging. Temporary symptom control could be achieved with an appendectomy.

## Introduction

Extramedullary hematopoiesis (EMH) is defined as the presence of hematopoietic tissue external to the bone marrow. Extramedullary hematopoiesis has been most commonly observed in the liver and spleen and sometimes in lymph nodes. The incidence of nonhepatosplenic extramedullary hematopoiesis (NHS-EMH) is rare and has been rarely reported [1]. The incidence of NHS-EMH occurring in the appendix is extremely rare, with only two cases having been previously reported, to the best of our knowledge [2,3]. 

Extramedullary hematopoiesis is associated with various underlying conditions, including primary myelofibrosis, thalassemia, hereditary spherocytosis, sickle cell disease, and acute leukemia [4, 5]. In terms of management, asymptomatic cases typically do not require intervention. However, symptomatic cases may lead to complications such as compression of surrounding structures, resulting in organ dysfunction, pain, or even rupture and bleeding, necessitating treatment [1]. We hereby present a rare case of appendiceal NHS-EMH and discuss its clinical presentation, diagnosis, and management with a literature review.

## Case presentation

A male patient in his 70s had been previously treated for follicular lymphoma seven years ago and was under regular follow-up. He gradually developed leukopenia. A bone marrow examination performed six months back confirmed a diagnosis of myelodysplastic syndrome with excess blasts 2 (MDS-EB-2). He was categorized as intermediate, based on the revised International Prognostic Scoring System (IPSS-R). A COVID-19 infection delayed the induction of azacitidine treatment.

**Figure 1:**
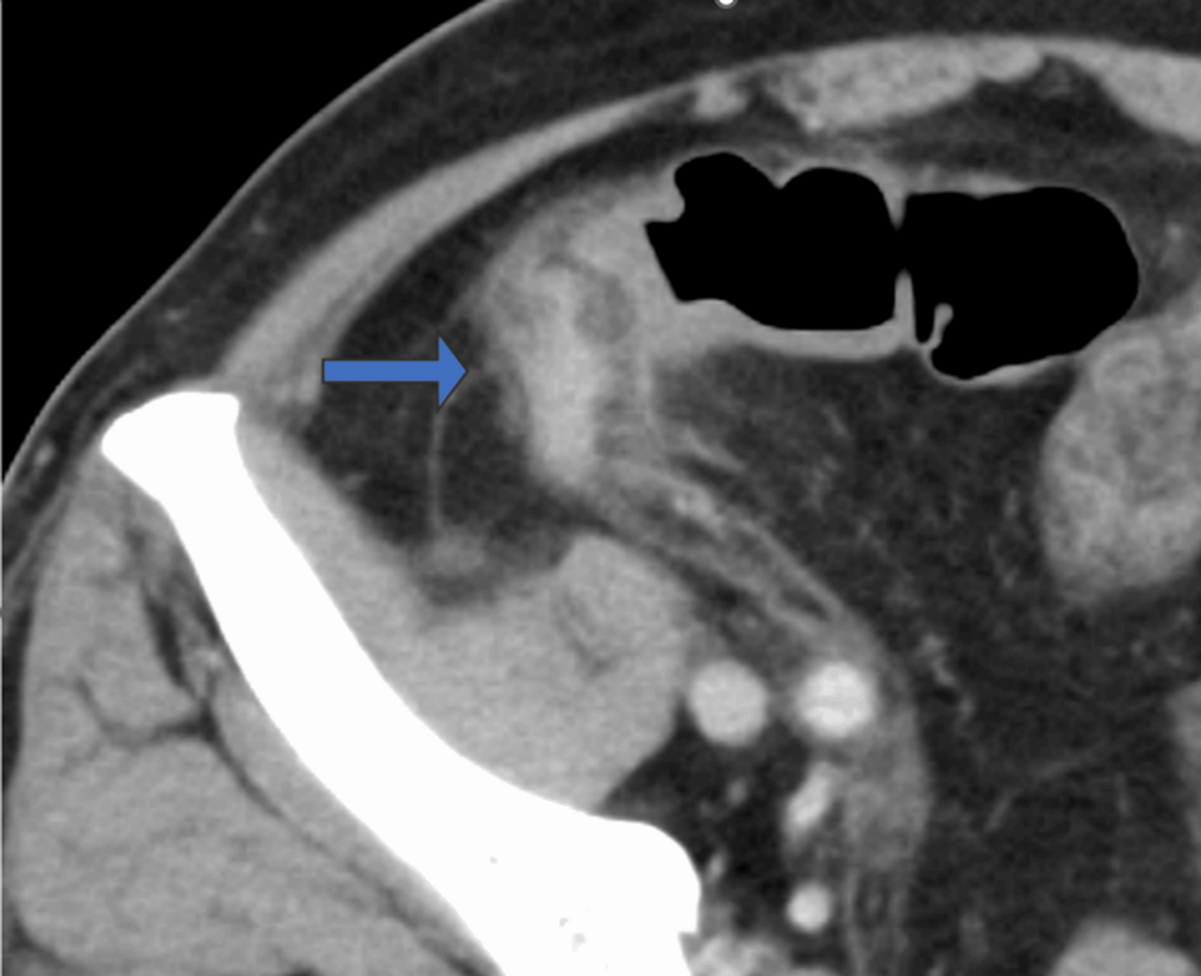
Contrast-enhanced CT revealed appendiceal wall thickening and periappendiceal fat stranding (blue arrow).

Four days prior to admission, the patient experienced generalized abdominal pain, which was later found to be localized to the right lower quadrant and was accompanied by fever and appetite loss. Physical examination revealed tenderness in the lower right quadrant. Laboratory tests revealed an elevation in the levels of inflammatory markers, with a C-reactive protein (CRP) level of 24.4 mg/dL (normal range: 0.00-0.14 mg/dL) and a procalcitonin (PCT) level of 31.2 mg/mL (normal range: 0-0.046 mg/dL). The white blood cell count was elevated to 16,470/μL (normal range: 3,300-8,6000/µL), while the hemoglobin level had reduced to 7.6 g/dL (normal range: 13.7-16.8 g/dL) and platelets to 144,000/µL (normal range: 158,000-348,000/µL). A diagnosis of acute appendicitis was made after abdominal CT imaging revealed thickening of the appendiceal wall and increased fat density around it (Figure 1). There was no evidence of hepatosplenomegaly.

**Figure 2:**
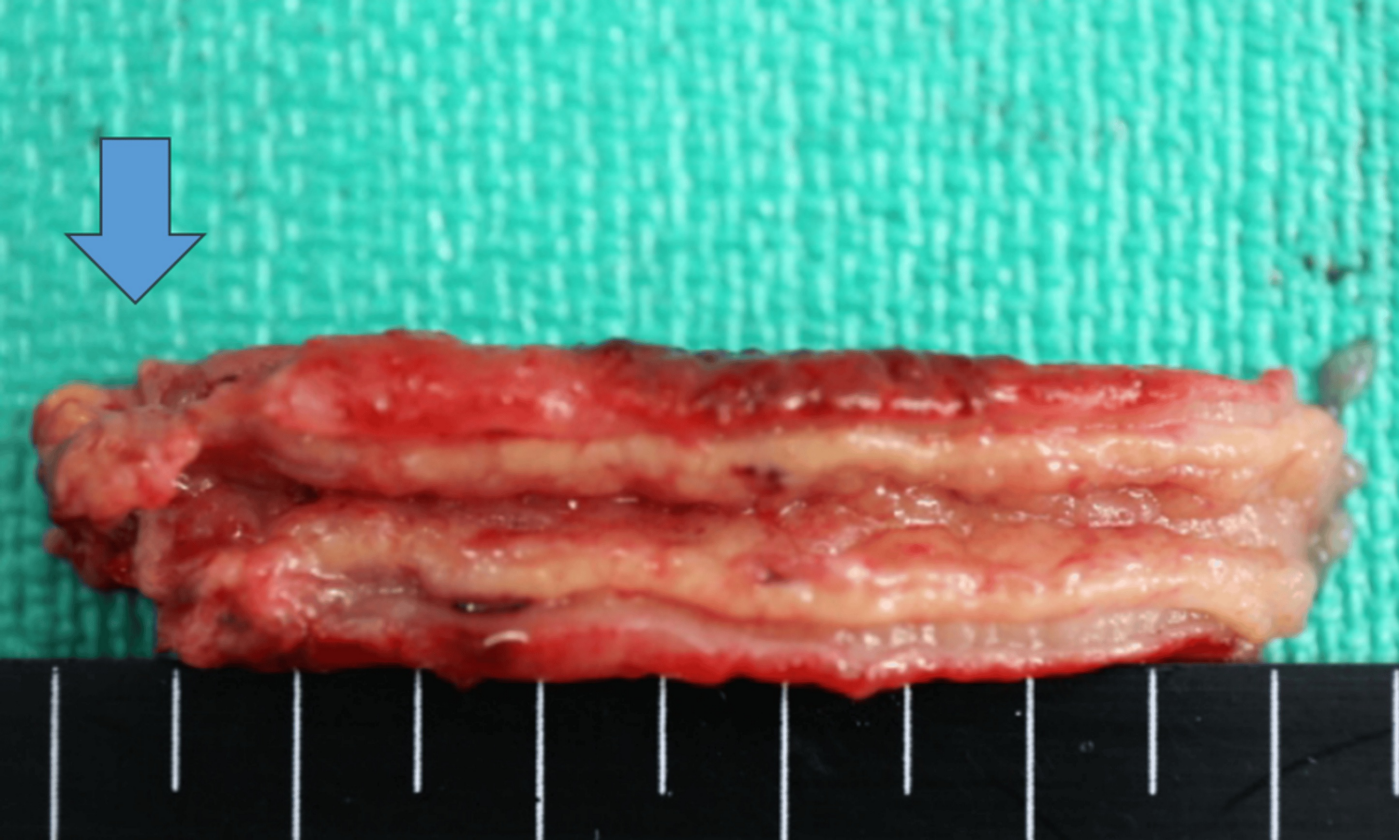
Macroscopically, the appendix was hyperemic with perforations at the tip (blue arrow).

The patient requested conservative management; however, his condition failed to improve, and emergency surgery had to be performed the following day. Laparoscopic examination revealed an inflamed appendix with hyperemia, with its tip being firmly attached to the greater omentum and sigmoid colon. Separation of the adhesions and subsequent drainage of the abscess revealed a perforation at the tip of the appendix. A marked inflammation of the appendix wall and surrounding peritoneum was consistent with localized peritonitis. Laparoscopic appendectomy was performed by dissecting the mesoappendix and stapling the appendiceal base using an automatic suturing device, and a drain was inserted. Macroscopically, the appendix was hyperemic with perforations at the tip (Figure 2).

**Figure 3:**
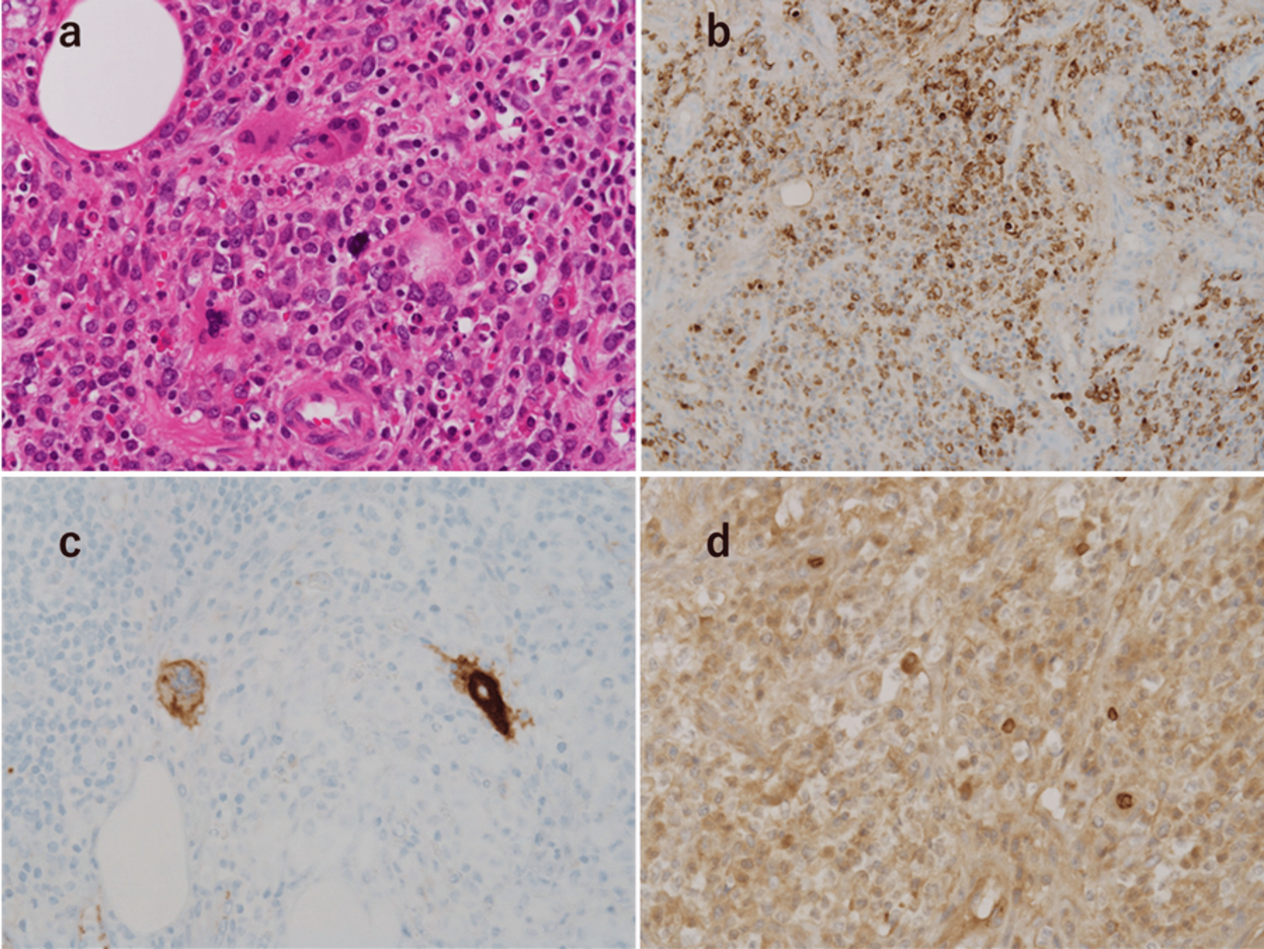
Extramedullary hematopoiesis, including granulocytes, megakaryocytes, and erythroblasts, was observed in the submucosal layer of the appendix Hematoxylin and eosin: ×40: (a) the cells are positive for b) MPO, c) CD61, and d) glycophorin C.

After an uneventful postoperative course, the drain was removed on the seventh postoperative day. The patient was discharged on the eighth postoperative day. Histopathological examination revealed inflammatory changes in the serosa; however, there was no evidence of acute appendicitis in the mucosa (Figure 3). A diffuse proliferation of round-shaped cells with mildly irregular nuclei was observed in the submucosal layer. Immunohistochemical staining was positive for myeloperoxidase (MPO), CD61, and glycophorin C, confirming the presence of granulocytes, megakaryocytes, and erythroblasts, leading to a diagnosis of appendiceal NHS-EMH.

There was no rapid progression of cytopenia postoperatively; however, four months later, his MDS progressed to acute leukemia. Despite treatment, his condition deteriorated, and he passed away 14 months following surgery.

## Discussion

Extramedullary hematopoiesis is an abnormal hematopoietic process that takes place outside the bone marrow in response to impaired marrow function. Extramedullary hematopoiesis most commonly affects the liver and spleen, and reports of NHS-EMH are relatively rare. Various sites in the body, including the paravertebral areas, heart, chest, thymus, kidneys, adrenal glands, prostate, pleura, skin, nerves, and the spinal canal can be affected by NHS-EMH, and it has also been reported to arise within tumors [1]. Involvement of the appendix is extremely rare, and only three cases have been reported to date, including the present case [2,3]. Primary myelofibrosis, thalassemia, hereditary spherocytosis, sickle cell disease, and acute leukemia are the underlying conditions associated with EMH [4,5].

Several hypotheses have been proposed to explain the pathogenesis of EMH. A few of them are as follows: (1) reactivation of embryonic hematopoietic organs, where tissues that exhibited hematopoietic activity during the fetal stage regain this function; (2) direct expansion of bone marrow into the adjacent tissues (para-marrow extension); (3) hematopoietic cells are transported via the bloodstream to distant sites in the phenomenon known as distal bone marrow embolism; and (4) transformation of multipotent stem cells into hematopoietic cells within specific organs [1]. Since the appendix is not an embryonic hematopoietic organ and is distant from the bone marrow, mechanisms (3) and (4) are the most plausible explanations for EMH in the current case.

Non-hepatosplenic extramedullary hematopoiesis is known to occur in lymph nodes, and the appendix is a tissue rich in lymphoid structures [6]. Although there is no direct evidence that these abundant lymph nodes contributed to the development of EMH in this case, their potential influence cannot be ruled out. While reports of appendiceal EMH are rare, it is conceivable that asymptomatic appendiceal EMH may coexist in patients with known EMH who present with appendiceal wall thickening detected in imaging studies.

The preoperative diagnosis of EMH is challenging because of its nonspecific presentation. Imaging modalities including CT, MRI, 18F-fluoro-3′deoxy-L-thymidine PET/CT, and 99mTc scintigraphy can be used for diagnosing EMH6 [7]. Biopsy provides a definitive diagnosis of EMH; however, it is associated with the risks of bleeding and organ injury and is not practical in all cases [8]. No hepatosplenomegaly was observed in this case; however, 82% of NHS-EMH cases present with hepatosplenomegaly, which can be helpful in diagnosing EMH [4].

Non-hepatosplenic extramedullary hematopoiesis is clinically significant due to its compensatory function for insufficient bone marrow hematopoiesis. Asymptomatic cases may not require interventions; however, symptomatic cases may develop complications such as organ dysfunction, pain, or rupture and bleeding, caused by the compression of surrounding structures, and require treatment [1]. Currently, there is no established consensus regarding the optimal treatment for NHS-EMH. Symptom management through radiotherapy or surgical intervention is the treatment option in cases where the underlying condition cannot be completely treated [9]. Hydroxyurea has also been reported as an effective treatment for EMH in patients with thalassemia [8, 10]. The treatment must be specifically designed after considering the affected site and the severity of the symptoms.

The two previously reported cases of appendiceal NHS-EMH followed a clinical course similar to that of acute appendicitis, making preoperative diagnosis of NHS-EMH challenging. However, the symptoms were resolved following an appendectomy. In our case, impaired hematopoiesis caused by MDS led to the development of extramedullary hematopoiesis in the appendix, which resulted in increased intraluminal pressure, subsequent perforation, and localized peritonitis. Nevertheless, symptoms improved after the appendectomy. Despite appendiceal NHS-EMH being a rare condition, our findings indicate that treating it like a typical acute appendicitis might be an effective approach for symptom control.

## Conclusions

Nonhepatosplenic extramedullary hematopoiesis is rare, and therapeutic intervention is necessary in the presence of complications. Currently, there is no established consensus regarding the optimal treatment for NHS-EMH, and it is essential to tailor the management based on the location and specific characteristics of NHS-EMH in each case.

This case is noteworthy due to the unique location of NHS-EMH. Appendiceal NHS-EMH is an extremely rare condition with clinical features similar to those of acute appendicitis, making preoperative diagnosis challenging. Appendectomy can be an effective option for managing complications such as perforation or abscess, as seen in this case.

## References

[1] Yang X, Chen D, Long H, Zhu B (2020). The mechanisms of pathological extramedullary hematopoiesis in diseases. Cell Mol Life Sci.

[2] Elpek GO, Bozova S, Erdoğan G, Temizkan K, Oğüş M (2006). Extramedullary hematopoiesis mimicking acute appendicitis: a rare complication of idiopathic myelofibrosis. Virchows Arch.

[3] Vasudevan JA, Nair RA, Prem S, Nair CK (2014). Co-existence of acute myeloid leukemia infiltration and extramedullary hematopoiesis in appendix. Indian J Pathol Microbiol.

[4] Koch CA, Li CY, Mesa RA, Tefferi A (2003). Nonhepatosplenic extramedullary hematopoiesis: associated diseases, pathology, clinical course, and treatment. Mayo Clin Proc.

[5] Ajayi F, Nali MS, Ali R, Patel A, Shaaban H (2022). Extra-medullary hematopoiesis in sickle cell disease presenting as a right adrenal mass. Cureus.

[6] Constantin M, Petrescu L, Mătanie C, Vrancianu CO, Niculescu AG, Andronic O, Bolocan A (2023). The vermiform appendix and its pathologies. Cancers (Basel).

[7] Zade A, Purandare N, Rangarajan V, Shah S, Agrawal A, Ashish J, Kulkarni M (2012). Noninvasive approaches to diagnose intrathoracic extramedullary hematopoiesis: 18F-FLT PET/CT and 99mTc-SC SPECT/CT scintigraphy. Clin Nucl Med.

[8] Karimi M, Cohan N, Pishdad P (2015). Hydroxyurea as a first-line treatment of extramedullary hematopoiesis in patients with beta thalassemia: four case reports. Hematology.

[9] Masalma R, Zidan T, Abualhumos KM (2024). Unraveling a rare case of epidural extramedullary hematopoiesis in a patient with transfusion-dependent beta thalassemia presenting with spinal cord compression. Cureus.

[10] Meo A, Cassinerio E, Castelli R, Bignamini D, Perego L, Cappellini MD (2008). Effect of hydroxyurea on extramedullary haematopoiesis in thalassaemia intermedia: case reports and literature review. Int J Lab Hematol.

